# Case Report: Streptococcus Suis Meningitis Diagnosed in a HIV-Infected Patient With Cryptococcal Meningitis Using Next-Generation Sequencing

**DOI:** 10.3389/fmed.2021.736064

**Published:** 2021-10-28

**Authors:** Yirui Xie, Bing Ruan, Guanjing Lang, Biao Zhu

**Affiliations:** State Key Laboratory for Diagnosis and Treatment of Infectious Diseases, National Clinical Research Center for Infectious Diseases, Collaborative Innovation Center for Diagnosis and Treatment of Infectious Diseases, The Department of Infectious Diseases, The First Affiliated Hospital, School of Medicine, Zhejiang University, Hangzhou, China

**Keywords:** next-generation sequencing, meningitis, streptococcus suis, cryptococcus, cerebrospinal fluid, HIV

## Abstract

**Background:** Streptococcus suis has been recognized as a zoonotic pathogen that may cause infections in humans. Although rarely described, it is not surprising that both cryptococcal and streptococcus suis meningitis infections can co-exist in a HIV-infected patient with a low CD4 count. However, a fast and accurate diagnose of meningitis of multipathogenic infections is challenging. In this report, we describe such a case of a HIV-infected patient with meningitis of multipathogenic infections.

**Case Presentation:** The patient was a 34-year-old Chinese male who was diagnosed with cryptococcal meningitis and HIV at the same time about 1 year ago. During the same time period, he had received (with good compliance) fluconazole and tenofovir-lamivudine- dolutegravir based antiretroviral therapy (ART). However, symptom of progressively worsening occipital headache appeared after he was exposed to a truck which was used for transporting pigs. Initial workup indicated an increase of the cerebrospinal fluid (CSF) opening pressure (OP) and an increase in the number of lymphocytes and proteins in CSF. A magnetic resonance imaging (MRI) scan revealed that partial cerebellar surface enhancement. The cryptococcus capsular antigen test of CSF was positive. The results of the India Ink microscopy for cryptococcus, nucleic acid of CMV and EBV and mycobacterium tuberculosis (MTB) tests of CSF were negative. The results of the bacteria and fungi smear and culture of CSF were also negative. Eventually, streptococcus suis was detected using next-generation sequencing (NGS) in CSF. The diagnosis of Streptococcus suis meningitis was made based on the patient's contact history with carrier pigs and the clinical findings addressed above. The treatment of 2 weeks of intravenous ceftriaxone and 1 week of oral moxifloxacin resulted in improvement of the condition of CSF. The anti-fungal treatment using fluconazole continued until the CFS OP went down to a normal level and the cryptococcus capsular antigen test of CSF was negative 6 months later.

**Conclusion:** This case highlights that NGS might be beneficial to HIV-infected patients who have meningitis with negative CSF culture results. Multiple etiologies for such condition in the immunocompromised patients must be taken into consideration and early stage NGS is recommended.

## Introduction

Compared to patients with monomicrobial meningitis, patients infected with multiple types of meningitis are more difficult to be diagnosed (1). However, a fast and accurate diagnosis of the pathogens causing the meningitis is challenging because of the limitations of current conventional tests (2). Sometimes, it is still difficult to draw the correct conclusion based on the test results. Streptococcus suis is a gram-positive, capsulated, hemolytic, facultative anaerobic coccus. It has recently been recognized as a zoonotic pathogen that may cause infections in humans in occupational contact with pigs and/or pork, with meningitis as its most common clinical presentation (2–5). Although rarely described, it is not of surprise that both cryptococcal and streptococcus suis meningitis infections can co-exist in a patient infected with human immunodeficiency virus (HIV) and with a low CD4 count. Next-generation sequencing (NGS), a method with highly sensitive detecting function for analyzing the microbiome, can provide additional valuable information for the detection of pathogens. Here, we present a report of a HIV-infected patient who had cryptococcal meningitis infection for about 1 year and was then diagnosed with streptococcus suis meningitis using NGS, even though his blood and CSF cultures test results were both negative.

## Case Report

**Figure 1:**
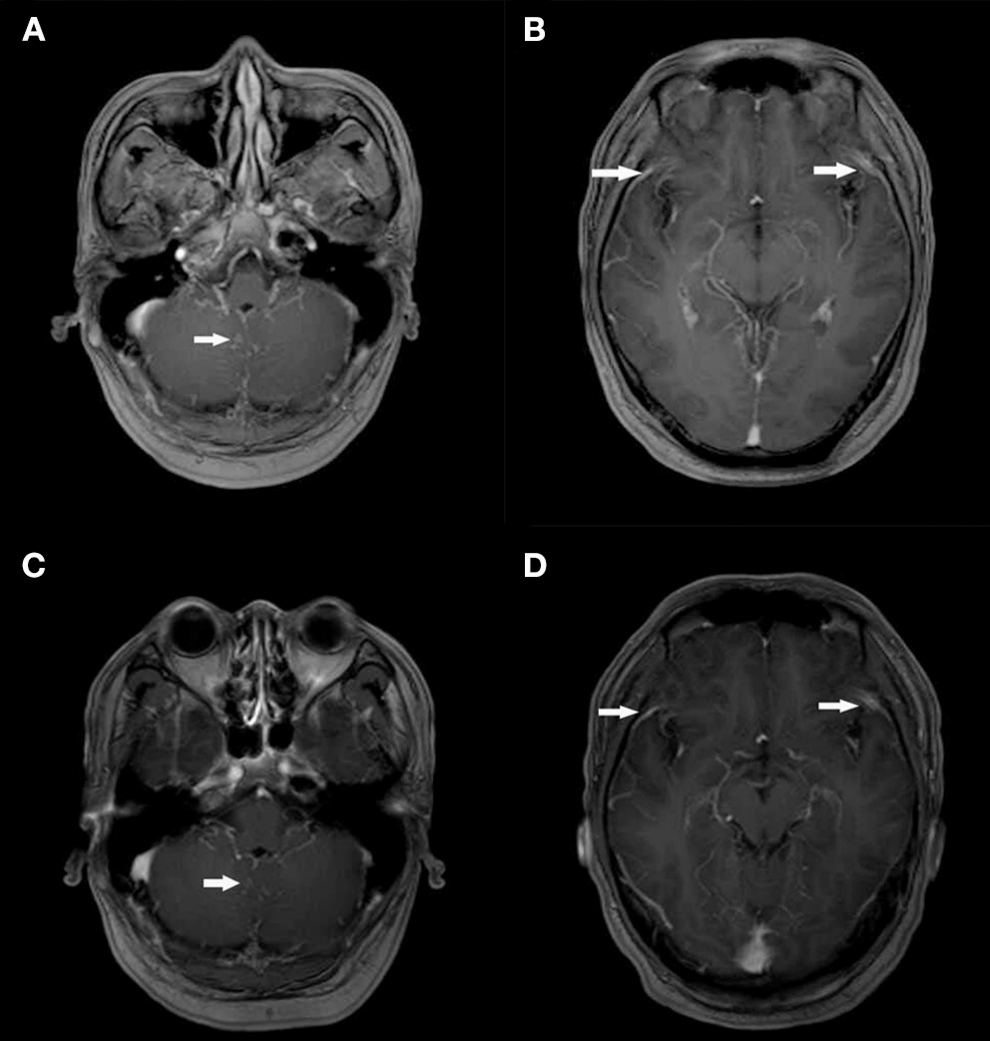
Contrast-enhanced and Diffusion-weighted Cranial MRI Scans on Day 3 of Admission and after 6 Months. On the third day of admission, post-contrast T1 MRI showed partial cerebellar surface enhancement **(A,B)** and get better at 6 months later after discharge **(C,D)**.

A 34-year-old Chinese male visited his local tertiary hospital due to progressively worsening occipital headache for the past 4 days, without showing any other symptoms of fever, projectile vomiting and so on. His medical history showed that he had had cryptococcal meningitis and HIV infection for about 1 year, and he had been receiving (with good compliance) fluconazole and tenofovir-lamivudine-dolutegravir based antiretroviral therapy (ART) during the same time period. The levels of the inflammatory markers were normal (Leukocytes 5.61^*^10^9^ cells/mL, C-reactive protein 7 mg/dL). His neurological signs showed no abnormal indications. Head Computed Tomography (CT) scan revealed no abnormalities. The lumber puncture was performed and the test result of CSF is shown in Table 1. It shows rising levels of OP, lymphocytes and protein in CSF. The cryptococcus capsular antigen test result of CSF was positive but the results for the gram staining and culture study were negative. The patient was diagnosed with cryptococcal meningitis and was given fluconazole (400 mg, QD) continuously and TMP-SMX (0.48, QD) for 1 week by the doctor at the local hospital. However, his headache problem was not resolved. Then he came to our institution, the Infectious Disease Department of the First Affiliated Hospital, School of Medicine, Zhejiang University, and was hospitalized. Upon arrival, the patient's body temperature was 36.7 C, pulse rate was 95 beats/min, blood pressure was 138/97 mmHg, respiratory rate was 23 breaths/min, and finger pulse SpO2 was 98%. He was suffering from headache but his neurological signs were normal. The laboratory results are as follows: white blood cell count 7.0 × 10^9^/L, with a neutrophil ratio of 42%, hemoglobin 150 g/L, and platelet count 294 × 10^9^/L; C-reactive protein (CRP)1.8 mg/L; CD4 count 171 cells/μL; normal blood biochemical; negative 1,3-beta-D-glucan test (G test) and galactomannan test (GM test) results (0.1 μg/L). The IgG antibodies of Epstein-Barr virus (EBV) and Human *Cytomegalovirus* (CMV) were positive, while the IgM antibodies and the test of the nucleic acid of EBV and CMV were negative. The test result for mycobacterium tuberculosis (MTB) was negative based on the blood test with T-SPOT, and the result for cryptococcus capsular antigen test of the blood was positive. The chest CT scan and echocardiography results were normal. The lumber puncture was performed on day 2 and the opening pressure was 285 (cm H2O). The test result of CSF is shown in Table 1. The cryptococcus capsular antigen test of CSF was positive. An India Ink microscopy on the CSF sample for cryptococcus was also performed and the result was negative. The results of the blood and CSF smears of bacteria and fungi were negative. The results of the acid-fast staining of CSF smear and GeneXpert were both negative for MTB. The test of the nucleic acid of CMV and EBV in CSF was negative. Based on the negative gram staining and culture study results of CSF provided by the local hospital and the above results, the CSF sample was sent to the laboratory for conventional tests and NGS test (using the Illumina Platform, IngeniGen. Ltd, Zhejiang, China) for pathogen-induced meningitis verification. Contrast-enhanced and diffusion-weighted MRI (3.0T) was performed on day 3, and the partial cerebellar surface enhancement was revealed (Figure 1), which matched the manifestation of cryptococcal meningitis, and no abnormal signals were detected in the cerebral parenchyma. Two days later, the NGS results showed that streptococcus suis (6 reads) and streptococcus mitis (1 reads) were found in CSF. The results of the blood and CSF culture of bacteria and fungi were still negative on day 5. So, we inquired the patient's medical history again, and he told us he had had close contact with a truck transporting pigs and had choked on water in a water park 2 weeks before his admission. Based on the test reports and his contact history, the diagnosis of streptococcus suis infection was made and intravenous Ceftriaxone (2.0, QD) was given. The patient's headache problem was resolved gradually. A second lumber puncture performed on day 12 revealed improved conditions of CSF (see Table 1), with negative gram staining and culture study results. Ceftriaxone was given to the patient for another 2 weeks and then oral moxifloxacin (0.4, QD) for 2 days. The third lumber puncture was performed on day 17, showing continuous conditional improvement of CSF. The patient was discharged in stable condition on day 21 after 1 week of receiving moxifloxacin. Three weeks later, we performed the fourth lumber puncture to confirm that the meningitis infection was cleared, but the patient's OP level was still abnormal, so fluconazole was given continuously until the OP level and cryptococcus capsular antigen test turned normal 6 months later.

**Table 1:** Cerebrospinal fluid profile evolution throughout treatment.

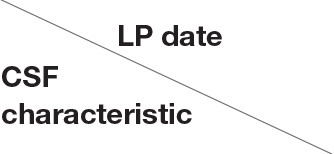	**2020.9.5 (Local hospital)**	**Day 2 2020.9.17**	**Day 12 2020.9.28**	**Day 17 020.10.3**	**Day 42 2020.10.28**	**Day 70 2020.11.25**	**Day 98 2020.12.23**	**Day 187 2021.03.22**
Opening pressure (cm H2O)	ND	285	250	210	220	220	210	140
Color	Colorless	Colorless	Colorless	Colorless	Colorless	Colorless	Colorless	Colorless
Turbidity	Clear	Clear	Clear	Clear	Clear	Clear	Clear	Clear
Cell count (/μL)	410	500	240	120	66	28	22	50
Neutrophils (%)	5	15	4	20	20			15
Lymphocytes (%)	90	80	92	80	80			80
Monocytes and others (%)		5	2					5
Protein (g/L)	ND	1.312	1.3172	1.471	0.753	0.675	0.629	0.401
Glucose (mmol/L)	3.1	2.7	2.7	2.5	2.7	3.7	3.3	4.2
Chlorine (mmol/L)	121	118	119	123	117	127	124	126
CrAg test	+	+	+	–	–	–	–	–
Lactate dehydrogenase (U/L)	ND	37	26	21	21	27	18	18
Adenylate deaminase (U/L)	ND	4.3	2.9	1.8	1.9	0.9	0.1	0.9
India Ink microscopy	–	–	–	–	–	–	–	–
CSF culture	–	–	–	–	–	–	–	–

*CSF, Cerebrospinal fluid; LP, Lumbar puncture; CrAg, Cryptococcal capsular polysaccharide antigen; ND, No data; “+”: Positive; “-”: Negative*.

## Discussion

The rate of AIDS-related opportunistic infections has decreased dramatically due to the success in achieving viral suppression and immune reconstitution combined with the effective and wide use of ART (6). The coexistence of fungal and bacterial meningitis in patients is rarely reported, so here we would like to address the case of the recent diagnosis of streptococcus suis meningitis using NGS in a patient who had prior diagnosed with HIV infection and cryptococcal meningitis about 1 year ago.

In the developed countries, the prevalence of cryptococcal meningitis has dropped amongst HIV patients as it can be diagnosed in the early stage, but the percentage is still exceedingly high in the more resource-limited environments (7). In this case, fluconazole was used and ART initiated 2 weeks later. The therapy went on for about 1 year and the patient's condition was stable but his cryptococcus capsular antigen test result of CSF was positive during the regular review. What's more, symptom of progressively worsening occipital headache appeared after the patient had contact with a truck which carried pigs. The routine blood and CSF test results were negative, and the diagnosis of streptococcus suis meningitis was finally confirmed using NGS on CSF.

Streptococcus suis has recently been recognized as a zoonotic pathogen that may cause infections in people with risk of occupational exposure to pigs and/or pork (3, 4). Meningitis is the most commonly resulted clinical presentation, followed by septicemia, endocarditis, arthritis, enteritis, spondylodiscitis, endophthalmitis, uveitis and peritonitis (3, 5). Streptococcus suis infection has no specific clinical symptoms in the early stage. Usually, Streptococcus suis meningitis starts with the typical presentation of meningeal symptoms such as fever, headache, nausea, and vomiting, as well as neurological symptoms, such as dizziness and balance disorder. The central nervous system imaging is the standard procedure in most cases. The diagnosis of purulent meningitis can be confirmed based on the CSF examination and the bacteria culture of CSF and blood (5). Typically, Streptococcus suis meningitis presented a neutrophilic CSF inflammatory response. However, this patient had cryptococcal meningitis infection and presented a lymphocytic CSF inflammatory response, hence clear conclusion could not be drawn in this regard. The patient's symptoms of headache and lymphocytic CSF inflammatory response might be caused by the co-occurrence of streptococcus suis meningitis and cryptococcal meningitis on top of his existing HIV infection and relatively low CD4 count. However, further investigation needs to be conducted toward the exact mechanism at work. The conventional culture has been used in the clinical process. However, these conventional tests have its limitations in terms of sensitivity, speed, and spectrum for pathogen detection (8). The routine blood and CSF culture results were negative in the two times of lumbar punctures conducted on the patient. So, a prompt and accurate diagnosis of meningitis of multipathogenic infections in HIV-infected patients is still difficult to achieve. NGS, as a highly sensitive, culture-independent and unbiased method, can identify all potentially known, new or unexpected pathogens (9, 10).

NGS has been successfully used as a diagnostic tool for various infectious diseases in the immunocompromised hosts (11–13). Previous study showed that multiple pathogens were identified by NGS in CSF from more than 15.9% (14/88) of HIV-infected patients who were suspected to have CNS infection with negative result by conventional tests. This indicated that multipathogenic CNS infections are not uncommon in people living with HIV and NGS is capable to identify multiple pathogens in a single test in an unbiased manner (1). Although there is one previous report about the application of NGS in the identification of streptococcus suis septicemia in a patient whose blood bacterial cultures results were negative (14), its application in diagnosing streptococcus suis meningitis in HIV-infected patients with cryptococcal meningitis history has never been addressed until now. Most patients with streptococcus suis meningitis respond well to treatment of broad-spectrum intravenous antibiotics such as penicillin, ampicillin and cefotaxime (5). Although the numbers of the NGS sequence read for streptococcus suis was low, the diagnosis of streptococcus suis meningitis was confirmed in this patient's case based on his contact history with carrier pigs and the clinical findings. The 2 weeks treatment of intravenous ceftriaxone and 1 week of oral Moxifloxacin resulted in the continuous improvement of the condition of CSF.

This case demonstrates that NGS might be beneficial to help diagnose HIV-infected patients who have meningitis with negative CSF cultures results and are more likely to be infected by multiple pathogens. Multiple etiologies for such condition in immunocompromised patients must be taken into consideration and early stage NGS is recommended. However, the application of NGS in clinical diagnosis is still challenging due to the consideration of the cost-effectiveness and the standardization of the entire procedure, from sample collection to result interpretation (15). The result interpretation of NGS requires caution, and other clinical, laboratory and radiological findings must be taken into consideration as well.

## Data Availability Statement

The original contributions presented in the study are included in the article/supplementary material, further inquiries can be directed to the corresponding author/s.

## Ethics Statement

Ethical review and approval was not required for the study on human participants in accordance with the local legislation and institutional requirements. The patients/participants provided their written informed consent to participate in this study.

## Author Contributions

YX collected the data and wrote the manuscript. GL and BZ analyzed and interpreted the patient data. BR and BZ reviewed the manuscript. All authors contributed to the article and approved the submitted version.

## Funding

This study was funded by the Natural Science Foundation of China (Young Scientist Fund, 81500491) and the National Science Foundation of China (Major Research Plan, 2018ZX10715-014). These funding agencies have no role in the design, data collection, analysis or interpretation of the research or in the writing of the manuscript.

## Conflict of Interest

The authors declare that the research was conducted in the absence of any commercial or financial relationships that could be construed as a potential conflict of interest.

## Publisher's Note

All claims expressed in this article are solely those of the authors and do not necessarily represent those of their affiliated organizations, or those of the publisher, the editors and the reviewers. Any product that may be evaluated in this article, or claim that may be made by its manufacturer, is not guaranteed or endorsed by the publisher.
